# Facile synthesis of robust Ag/ZnO composites by sol–gel autocombustion and ion-impregnation for the photocatalytic degradation of sucrose

**DOI:** 10.1038/s41598-023-39479-7

**Published:** 2023-07-27

**Authors:** Lalita Buengkitcharoen, Sittipong Amnuaypanich, Suriyabhorn Naknonhan, Sirinuch Loiha, Nopbhasinthu Patdhanagul, Ammarika Makdee, Sujitra Amnuaypanich

**Affiliations:** 1 Department of Chemistry and the Center of Excellence for Innovation in Chemistry (PERCH-CIC), Faculty of Science, Khon Kaen University, Khon Kaen, 40002 Thailand; 2 Materials Chemistry Research Center (MCRC-KKU), Faculty of Science, Khon Kaen University, Khon Kaen, 40002 Thailand; 3 Department of General Science, Faculty of Science and Engineering, Kasetsart University, Sakon Nakhon, 47000 Thailand; 4 Synchrotron Light Research Institute, 111 University Avenue, Muang District, Nakhon Ratchasima, 30000 Thailand

**Keywords:** Chemistry, Materials science

## Abstract

Metallic Ag nanoparticles decorated on ZnO photocatalysts were prepared by facile sol–gel autocombustion followed by ion-impregnation. Electron microscopy studies revealed the presence of impregnated Ag as nanoparticles on ZnO surfaces, which affected the microstructure of ZnO particles. XRD patterns of Ag/ZnO composites confirmed the metallic phase of Ag. No peak shift for ZnO phase peaks suggests that the impregnated Ag was barely incorporated into the ZnO lattice. Consequently, DRS spectra of Ag/ZnO composites revealed the same absorption edges and *E*_g_ for pure and Ag/ZnO. The photocatalytic activity of Ag/ZnO composites for sucrose degradation under UV light was 40% higher than that of pure ZnO. Metallic Ag nanoparticles on the ZnO surface suppressed the surface defects and the recombination of photoexcited electrons and holes. The highest activity with 100% degradation of 100 ppm sucrose (1200 µg of carbon) within 105 min was achieved using ZnO with 10% w/w Ag (10% Ag/ZnO). Ag L3-edge XANES spectra of fresh and spent Ag/ZnO catalysts confirmed the stability of metallic Ag after the usage. The Ag/ZnO catalyst could be used for 5 cycles without losing photocatalytic activity. The Ag/ZnO catalyst was utilized to degrade sugar-contaminated condensate from the sugar mill. 10% Ag/ZnO revealed the highest photocatalytic performance, capable of degrading 90% of sugar in the condensate within 90 min.

## Introduction

In sugar manufacturing, the condensate generated from the condensation of juice vapor is usually contaminated with 40–50 ppm of sugars, mainly sucrose. Reuse of the condensate for process water, e.g., a boiler make-up water, cannot be made without removing contaminated sugars. Biological treatment methods (anaerobic and aerobic) are typically employed in sugar manufacturing due to their simplicity and low cost. However, the biological methods require a large treatment area, e.g., the wetlands, and take several weeks for complete degradation^1^. Hence an alternative method for treating the condensate with a higher rate of sugar degradation under a compact treatment unit is sought. It is known that the photocatalytic degradation technique is one promising method for removing organic pollutants in water. ZnO is the most practical use as a UV photocatalyst. However, the photocatalytic activity of ZnO is hindered by the high recombination rate of the electron and hole. The addition of metal nanoparticles such as Ag, Pt, Pd, Ni, and Cu on ZnO can reduce electron–hole recombination and improve photocatalytic activity^2,3^. Among those metals, metallic Ag presented higher performance due to the chemical stability of metallic Ag in aqueous conditions and is easily reduced into the metallic form^4^. Therefore, Ag-doped ZnO has been widely studied as a photocatalyst for the degradation of organic pollutants^5–7^. Several approaches have been employed to prepare high-performance Ag-doped ZnO photocatalysts, such as co-precipitation, hydrothermal, solvothermal, and sol–gel^8–11^.

In this work, the composites of metallic Ag nanoparticles decorated on ZnO were prepared by facile sol–gel autocombustion and ion-impregnation. The impregnation of Ag^+^ ion on calcined ZnO aims to limit the incorporation of Ag into the crystal lattice of calcined ZnO, which alters the chemical properties of host ZnO such as the amounts of defects in ZnO, band gap energy, unit cell parameters, etc. The temperature that can perform the thermal decomposition of Ag_2_O to metallic Ag was considered from the well-known Ellingham diagrams^12^. The correlation between the Gibbs free energies of the formation of metal oxide and temperature suggests that the metallic phase of Ag easily emerged at a temperature higher than 400 °C^13–15^ Under a fast-cooling rate in a closed space, the reoxidation of metallic Ag with oxygen during the cool-down step is limited. The role of metallic Ag nanoparticles on the surface of ZnO particles is to reduce the recombination of photoinduced electron–hole pairs in ZnO, increase the kinetic of interfacial charge transfer and enhance the photocatalytic activity.

## Results and discussion

### Microstructure and specific surface area of ZnO and Ag/ZnO

**Figure 1:**
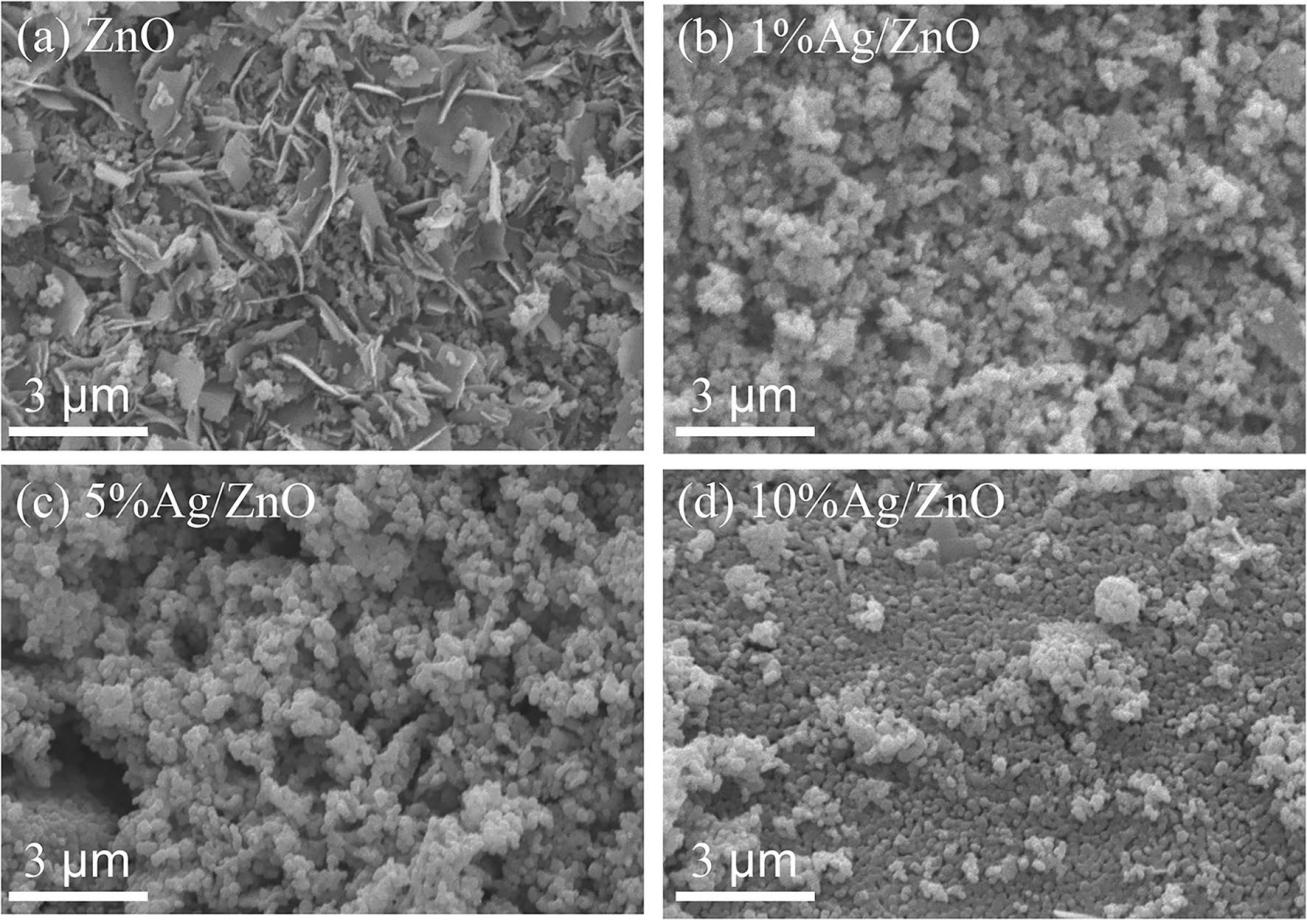
SEM micrographs of the (**a**) pure ZnO, (**b**) 1% Ag/ZnO, (**c**) 5% Ag/ZnO, and (**d**) 10% Ag/ZnO.

The SEM images of ZnO and Ag/ZnO in Fig. 1 manifest the change in the microstructure of calcined ZnO with the addition of Ag contents. The microstructure of unmodified ZnO consisted of thin crystalline plates and small round-shape particles with a diameter of less than 0.3 µm. The thin plates disappeared with adding Ag; therefore, only small particles are observed. The microstructure of ZnO with 1 wt% Ag and 5 wt% Ag reveals the agglomeration of round-shape particles with a particle size larger than that of ZnO. The disappearance of thin plates and the increase in particle size was due to the presence of Ag at ZnO grains which assists the liquid phase sintering and enhances the growth of particles^16^. This behavior of liquid phase sintering is pronounced in the 10 wt% Ag/ZnO sample. When increasing the Ag content to 10 wt%, the particles change from loosely agglomerated particles to well-connected particles with flattened contacts, so-called “necks”^17^. The appearance of the necks between particles confirmed that the particles were in the intermediate stage of the liquid sintering process during the calcination at 550 °C. It was found that the grain size of 10% Ag/ZnO was smaller than that of 5% Ag/ZnO, which is due to the limited solubility of Ag^+^ into the ZnO lattice by the size difference between Ag^+^ and Zn^2+^. Therefore, metallic Ag nanoparticles were deposited at grain boundaries which possess lower surface energy than grain surface. The deposition of Ag nanoparticles significantly hinders the growth of ZnO grains^16^. The sintering of ZnO particles in 10 wt% Ag/ZnO may improve the photocatalytic activity as the photoexcited electron and hole can easily transport between the connected grains. Therefore, the recombination rate is expected to decrease. In addition, the presence of Ag metal at the grain boundary also acts as a trapping site for electrons and promotes photocatalytic activity.

The BET specific surface areas of ZnO and Ag/ZnO samples evaluated from the N_2_ adsorption–desorption method revealed that ZnO and Ag/ZnO catalysts possessed low specific surface areas with the values of 6.44, 3.95, 6.83, and 4.63 m^2^/g for ZnO, 1% Ag/ZnO, 5% Ag/ZnO, and 10% Ag/ZnO, respectively. As observed in SEM, the change in microstructure slightly affected the specific surface area of the catalysts. The disappearance of thin plates in 1% Ag/ZnO causes a decrease in BET specific surface area. When Ag content increased to 5 wt%, the microstructure completely changed to round particles, slightly increasing the specific surface area. However, adding Ag content to 10 wt% causes the sintering between ZnO particles leading to a decrease in the specific surface area.

**Figure 2:**
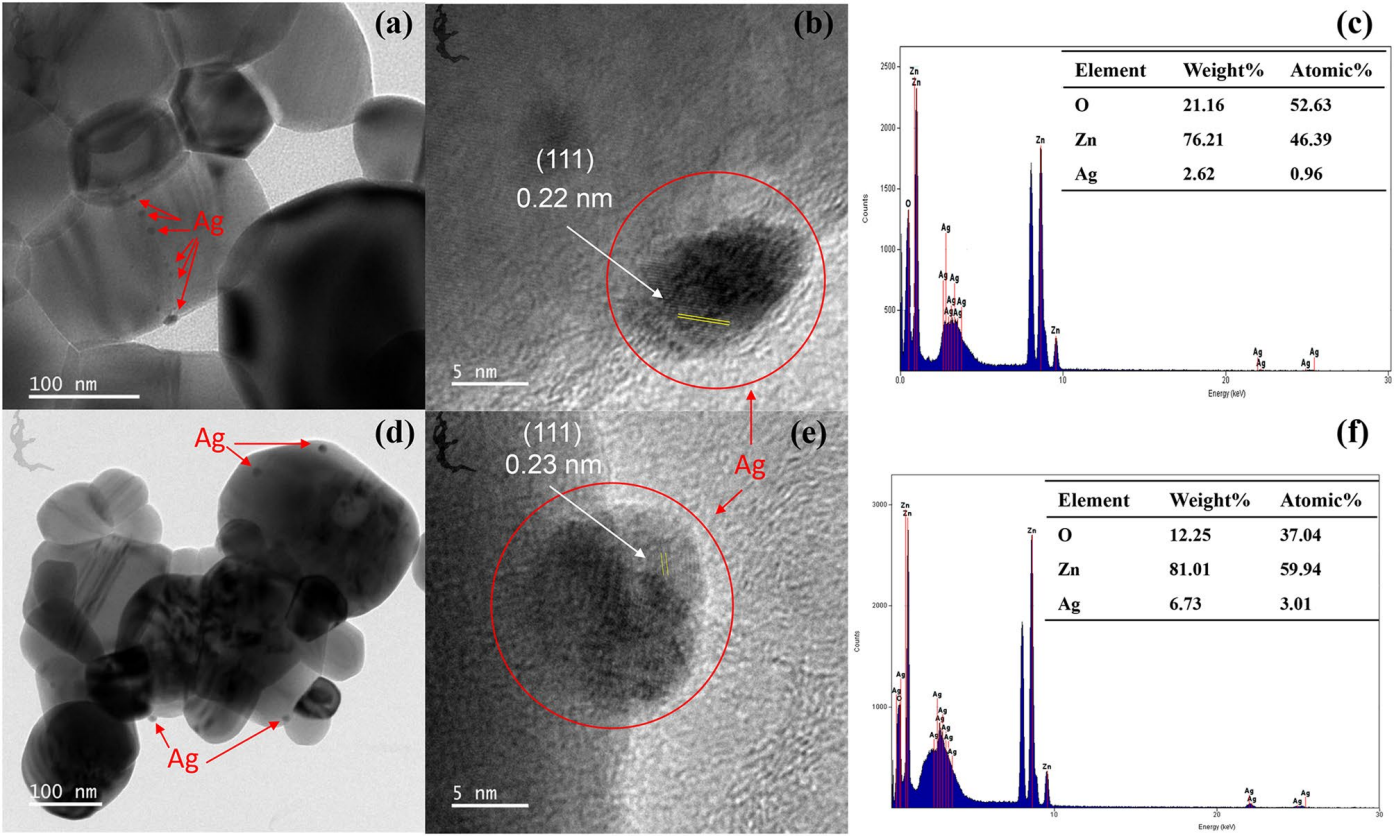
(**a**,**d**) TEM images, (**b**,**e**) HRTEM, and (**c**,**f**) EDS spectra of 5% Ag/ZnO and 10% Ag/ZnO, respectively.

TEM images of 5% Ag/ZnO and 10% Ag/ZnO shown in Fig. 2 reveal dense, round-shaped Ag nanoparticles depositing on the ZnO surface. EDS spectra in Fig. 2c and f confirmed the existence of Ag on ZnO, with the detected amount increasing with the amount of Ag incorporated into Ag/ZnO. The appearance of Ag nanoparticles at the neck between particles in Fig. 2a suggests that Ag is involved in the sintering of ZnO via the liquid phase sintering process. The high-resolution TEM (HRTEM) images in Fig. 2b and e display lattice fringes with an interplanar spacing of 0.22 nm corresponding to the (111) plane of metallic Ag^18^.

### Phase characterization

**Figure 3:**
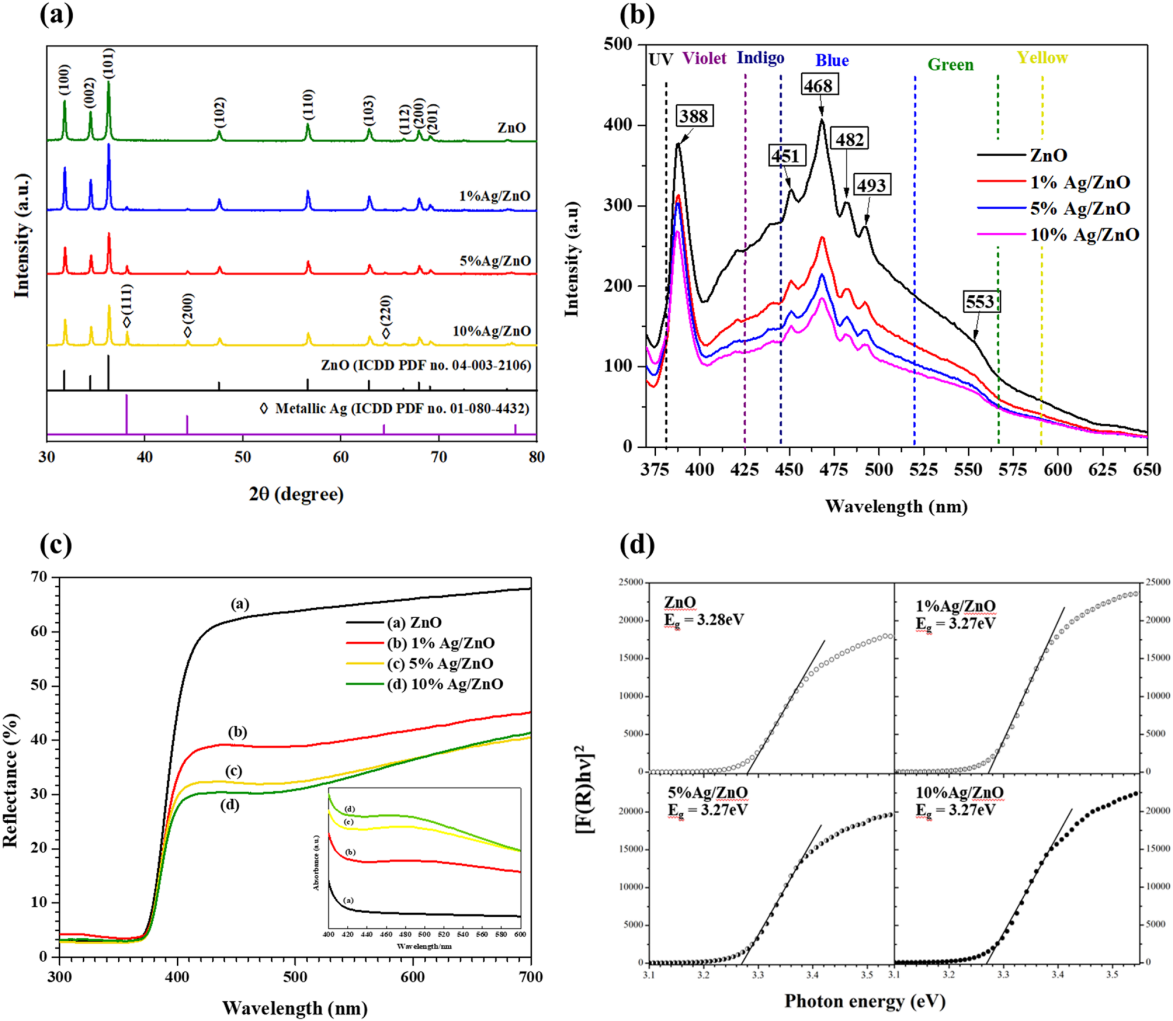
(**a**) XRD patterns, (**b**) PL spectra, (**c**) UV–Vis diffuse reflectance spectra, and (**d**) Tauc plots of pure ZnO with various Ag/ZnO catalysts.

The XRD patterns of ZnO and Ag/ZnO samples are presented in Fig. 3a. Ag/ZnO spectra contained a mixed phase of ZnO with hexagonal wurtzite phase (ICDD PDF no. 04-003-2106) with diffraction peaks at 2θ of 31.8°, 34.5°, 36.3°, 47.6°, 56.7°, 62.8°, 66.4°, 67.9° and 69.2° indexed to (100), (002), (101), (102), (110), (103), (200), (112) and (201) diffractions, respectively and metallic Ag with face-centered-cubic phase (ICDD PDF no. 01-080-4432)^19^ at 2θ = 38.2°, 44.4°, and 64.5° corresponding to (111), (200), and (220) diffractions, respectively. When increasing the Ag content, the intensity of ZnO peaks decreases because the incorporation of Ag^+^ ions in the ZnO lattice affects the crystallinity of the ZnO crystal structure. However, there is no significant peak shift of ZnO peaks in Ag-containing samples. The appearance of metallic Ag peaks with significant intensities indicates that Ag^+^ ions incorporate in the ZnO lattice with a limited amount since the ionic radius of Ag^+^ (1.22 Å) is higher than Zn^2+^ (0.74 Å) and the excess amount of Ag^+^ underwent thermal reduction during calcining temperature and primarily deposited on ZnO surface.

### DRS analysis

The optical band gap of pure ZnO and Ag/ZnO samples was assessed from UV–Vis DRS spectra shown in Fig. 3c. Both pure ZnO and Ag/ZnO comprise a main strong absorption edge at about 390 nm in the UV region. In Ag/ZnO samples, a new absorption peak at 470 nm appears, indicating the characteristic surface plasmon resonance (SPR) of metallic Ag nanoparticles^20^. The intensity of this visible-light absorption peak increases with increasing the Ag contents while the %R in this region decreases. Tauc plots in Fig. 3d were derived from the Kubelka–Munk function, (F(R_∞_) by (F(R_∞_)*h*ν)^1/γ^ = B(*h*ν − E_g_) where *h* is the photon energy, ν is the photon’s frequency, E_g_ is the band gap energy, B is constant and R_∞_ is the reflectance of an infinitely thick specimen, R_sample_/R_standard_^21^. In case of direct band gap ZnO, the γ factor is equal to 1/2^22^. The results reveal the unchanged band gap energy (E_g_) between pure ZnO and Ag/ZnO samples. These results are consistent with the results from XRD patterns and TEM images. Most metallic Ag nanoparticles prepared by the ion-impregnation method deposit on the ZnO surface and barely incorporate into the lattice of ZnO.

### Photoluminescence spectra

Figure 3b depicts the room temperature photoluminescence (PL) spectra of ZnO and Ag/ZnO composites with an excitation wavelength of 350 nm. The spectra comprise several emission bands, including a sharp and strong UV emission band at 388 nm (3.20 eV), a strong blue emission at 468 nm (2.65 eV), and weak emissions at 451 nm (2.76 eV), 482 nm (2.57 eV) and 493 nm (2.51 eV). The UV emission band arises from the direct recombination of the free excitons and is located close to the absorption edge of ZnO, known as a near-band edge (NBE) excitonic emission^23,24^. The visible luminescence peaks are associated with exciton transitions and defect emissions, generally called deep-level emissions (DLE). These emissions depend on ZnO's synthesis technique, morphology, and surface defects. The visible peaks are mainly blue emissions resulting from the presence of interstitial zinc (${Zn}_{i}^{..}$), zinc vacancy (${V}_{Zn}^{{\prime}{\prime}}$) and oxygen vacancy (${V}_{O}^{..}$) that involve the transition of photoexcited electrons to the other defects with lower energy level or the holes in the valence band^25^. The presence of Ag on the ZnO surface significantly decreased the intensity of all emission peaks. The quenching of all emissions is due to the direct electron transfer of photoexcited electrons from the conduction band of ZnO to the Fermi level of Ag with lower energy position^26^.

### Photocatalytic activity of Ag/ZnO

**Figure 4:**
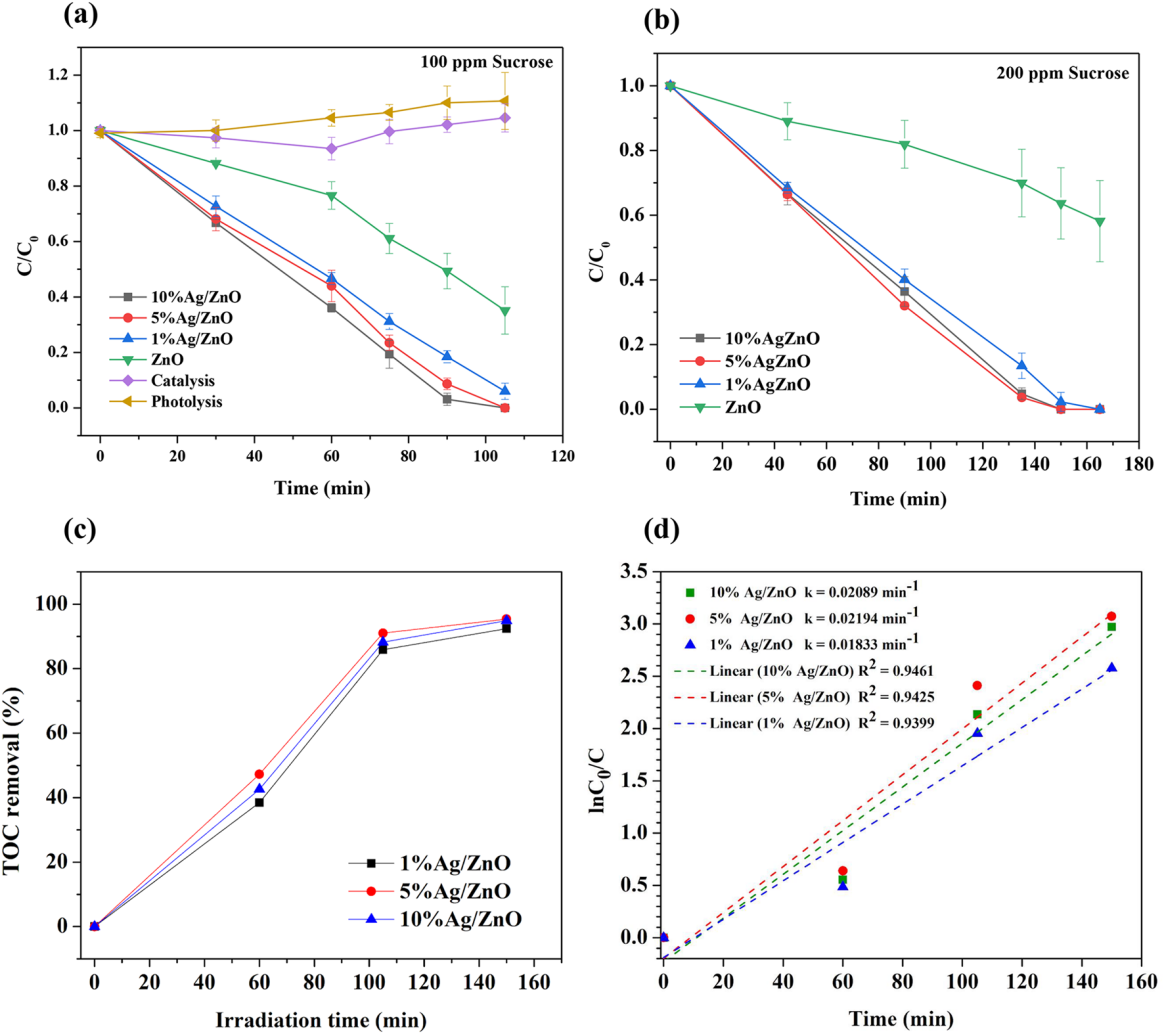
Photocatalytic degradation of pure ZnO and Ag/ZnO catalysts (**a**) 100 ppm sucrose (**b**) 200 ppm sucrose measured by UV–visible spectroscopy, (**c**) TOC removal of 100 ppm sucrose solution, and (**d**) Chemical kinetics of the photocatalytic degradation of sucrose over Ag/ZnO catalysts.

In Fig. 4a, Ag/ZnO catalysts demonstrated higher photocatalytic activity than pure ZnO by about 40%. For an initial 100 ppm of sucrose, the increase of Ag contents slightly increases the activity, and 10% Ag/ZnO showed the highest photocatalytic activity. The catalysis and photolysis experiments confirm that the degradation of sucrose occurs by the photocatalyst under UV light. When performing the experiment in higher sucrose concentration at 200 ppm (see Fig. 4b), the photocatalytic activities of all Ag/ZnO catalysts were superior to that of ZnO by approximately 60%. The activities of 5% Ag/ZnO and 10% Ag/ZnO were higher than those of 1% Ag/ZnO. The comparable photocatalytic activity of Ag/ZnO composites, regardless of the Ag content, was consistent with the previous study of Zheng et al.^18^. The slight increase of the photocatalytic activity with the increase of Ag content comes from the aggregation of Ag rather than evenly distributed thus larger Ag particles were found in TEM images of 10%Ag/ZnO compared with 5% Ag/ZnO.

### Total organic carbon (TOC) removal

The TOC in the degraded sucrose solution obtained from each Ag/ZnO catalyst confirms that almost 95% of sucrose and its derivatives were degraded within 150 min, as presented in Fig. 4c. Evaluating the overall rate of sugar degradation (sucrose and degraded products) from the TOC data, the degradation rates increase with the increase of Ag contents, as displayed in Fig. 4d. However, 5% Ag/ZnO and 10%Ag/ZnO catalysts showed a comparable rate of 0.021 min^−1^.

### Photocatalytic degradation mechanism

#### HPLC measurements

**Figure 5:**
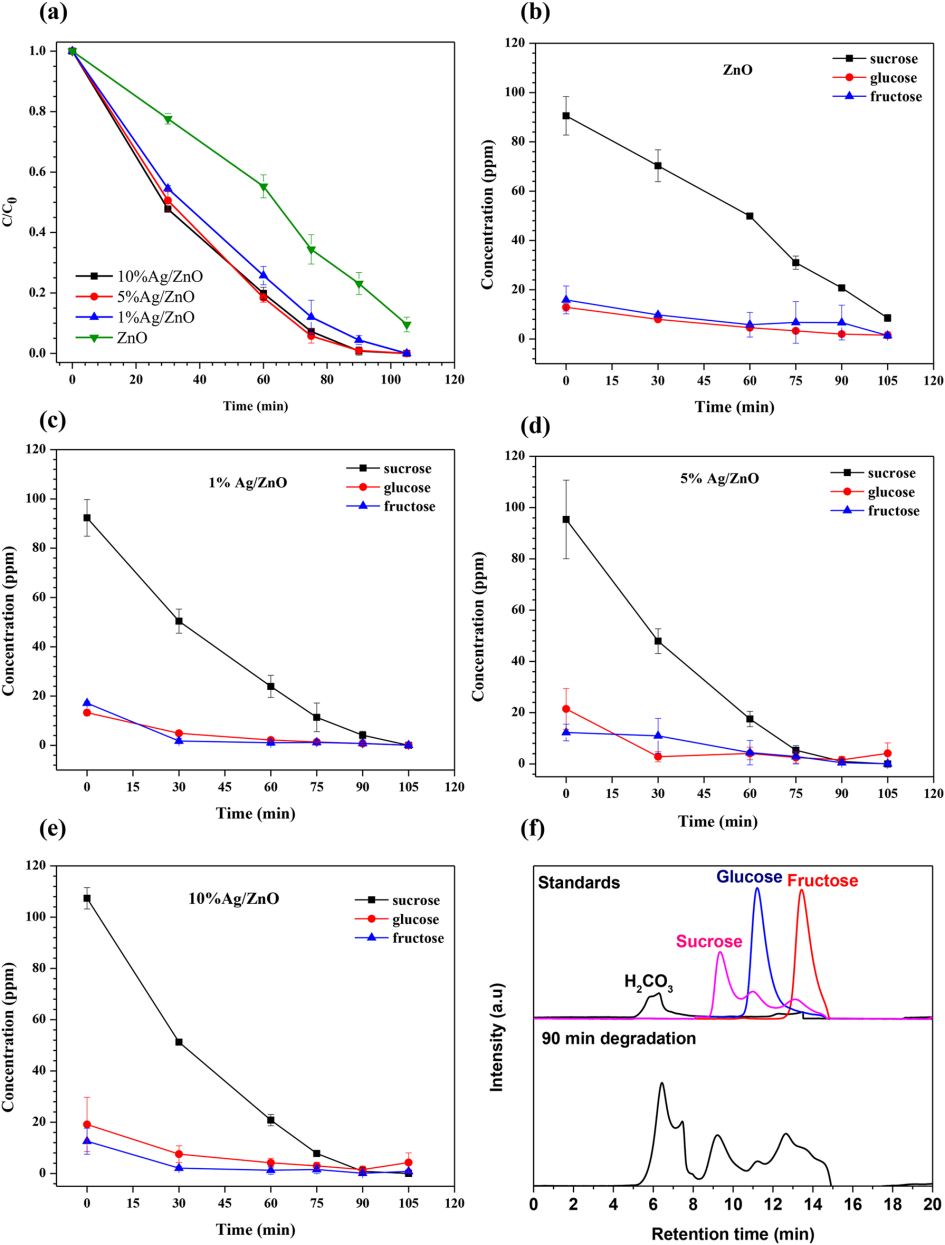
(**a**) Photocatalytic degradation of sucrose measured by HPLC, (**b**)–(**e**) The changes in concentration of sucrose, glucose, and fructose using pure ZnO and Ag/ZnO catalysts, (**f**) HPLC chromatograms of degraded sucrose solution over 10%Ag/ZnO catalyst at 90 min compared with standards.

The mineralization and degradation mechanism of sucrose were investigated by the HPLC technique. The sucrose solution at a specific time of the photocatalytic degradation was determined. The concentrations of sucrose, glucose, and fructose were determined from the calibration curves of those standard solutions. The degradations of sucrose evaluated from *C*/*C*_0_ vs. time graphs of pure ZnO and Ag/ZnO catalysts are presented in Fig. 5a. All Ag/ZnO catalysts have higher photocatalytic activity than pure ZnO and can degrade about 50% of sucrose within 30 min of UV irradiation. Hence the addition of metallic Ag on ZnO via the ion-impregnation method dramatically enhances the photocatalytic activity of ZnO.

Figure 5b–e reveals the change in concentrations of sucrose, glucose, and fructose changed during the photodegradation using pure ZnO and Ag/ZnO catalysts. In all samples, small amounts of glucose and fructose were detected in fresh sucrose solution (0 min). Within 30 min of irradiation, the concentrations of sucrose decreased by 50% of their initial amount, but those of glucose and fructose decreased to 90% and 75%, respectively. After 60 min, sucrose concentration declined proportionally, while glucose and fructose slightly decreased toward 0 ppm.

Figure 5f demonstrates the HPLC chromatogram after 90 min of degradation time. Apart from sucrose, glucose, and fructose peaks, the early peaks at a retention time of around 6 min were found after 30 min of degradation as presented in the Supplementary Information Fig. S1a. This peak belongs to the final degraded products, i.e., dissolved carbon dioxide (CO_2_) forming H_2_CO_3_, HCO_3_^−^, CO_3_^2−^ anions confirming with H_2_CO_3_ solution (pH 4.45) prepared by dissolution of gaseous CO_2_ in deionized water. These final degraded products of sucrose degradation were also reported by Penpolcharoen et al.^27^. This also explains the small amount of remaining carbon in the final solution examined by TOC. Therefore, it can be justified that the photocatalytic degradation of sucrose occurs via the degradation of sucrose to glucose and fructose, followed by the degradation of glucose and fructose to small organic acids and anions.

#### XANES spectra

**Figure 6:**
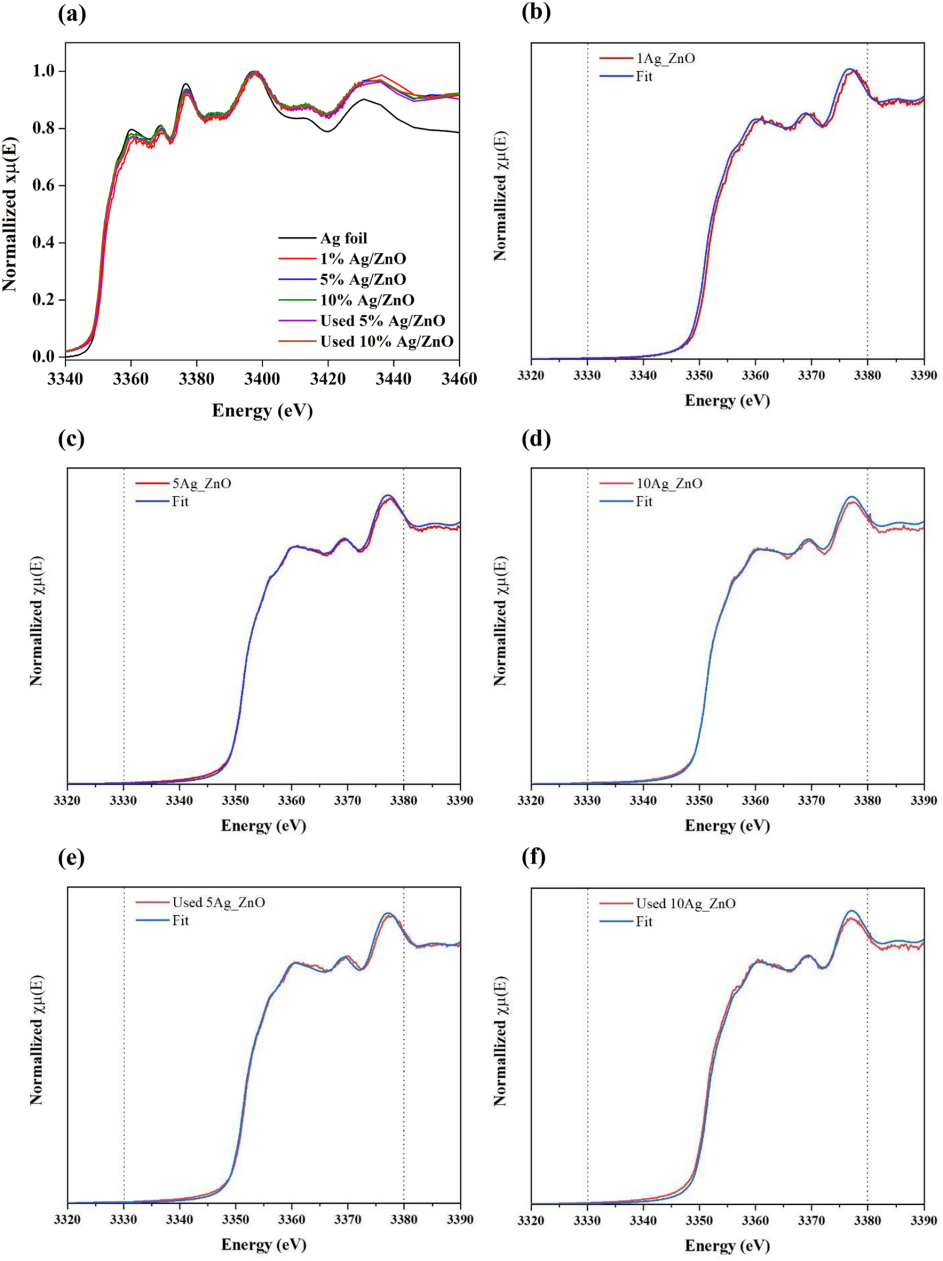
(**a**) XANES spectra of fresh and used Ag/ZnO catalysts compared with Ag foil and (**b**)–(**f**) linear combination fit results of the XANES Ag L3-edge.

The oxidation states of Ag in fresh 1, 5 and 10%Ag/ZnO and spent 5 and 10% Ag/ZnO were investigated by X-ray absorption spectroscopy (XAS). The Ag L3-edge XANES spectra in Fig. 6a of fresh and spent Ag/ZnO samples indicates that the oxidation state of Ag in all Ag/ZnO composites was zero. To confirm the oxidation state of silver on ZnO catalysts, linear Combination Fit (LCF) of XANES Ag L3-edge were calculated using the Athena package included in the IFEFFIT program. Three standards of Ag foil, Ag_2_O and AgO corresponding to Ag^0^, Ag^+^ and Ag^2+^, respectively were used. The component percentages of each species are reported in Table 1 and XANES fitting results are demonstrated in Fig. 6b–f. The Ag species on fresh and spent catalysts was zero valence with the main component of over 90% Ag^0^ and the minor component was Ag^+^. For the fresh catalysts with higher silver loading, the component of Ag^+^ became higher. However, after photocatalytic reaction, only Ag^0^ species were found on 5% and 10% Ag/ZnO. The disappearance of Ag_2_O in spent Ag/ZnO confirms the reduction of Ag_2_O to Ag^0^ via the transfer of photoexcited electrons from the conduction band of ZnO to Fermi level of Ag.

**Table 1:** Linear Combination fit of the XANES Ag L3-edge of fresh and used catalysts.

Sample	Component percentage (%)		
	Ag foil (Ag^0^)	Ag_2_O (Ag^+^)	AgO (Ag^2+^)
1%Ag/ZnO	100.0	0.0	0.0
5%Ag/ZnO	96.4	3.6	0.0
10%Ag/ZnO	92.3	7.6	0.0
Spent 5%Ag/ZnO	100.0	0.0	0.0
Spent 10%Ag/ZnO	100.0	0.0	0.0

#### Scavenger experiment

**Figure 7:**
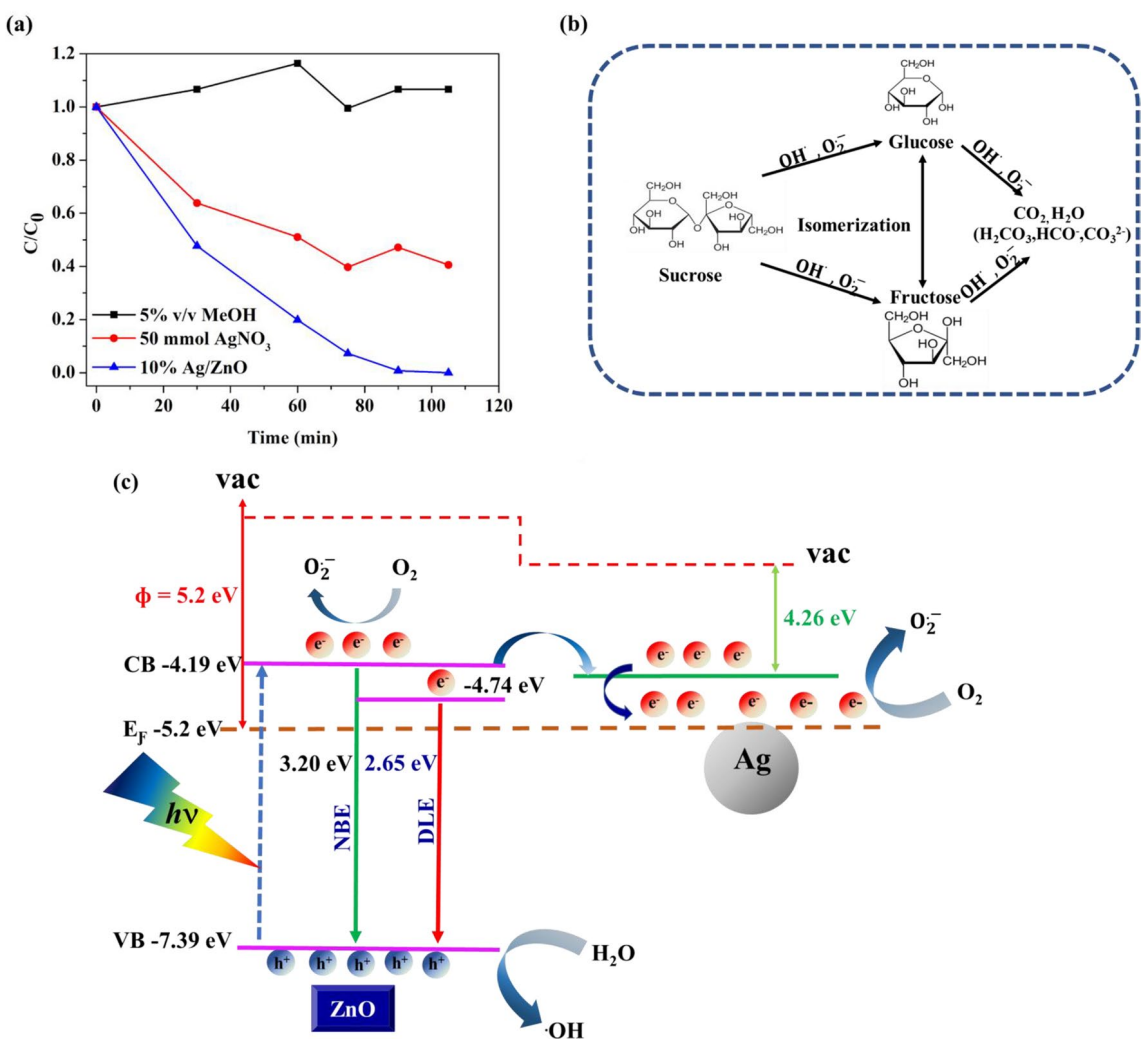
(**a**) Scavenger experiment of active species during the photocatalytic degradation of sucrose over 10%Ag/ZnO under UV light irradiation and (**b**) schematic photocatalytic mechanism of Ag/ZnO catalyst. (**c**) Proposed energy band diagram of Ag/ZnO catalysts.

The active species involved in the photocatalytic degradation of sucrose over Ag/ZnO catalyst were investigated by adding an electron scavenger (AgNO_3_) and hydroxyl radical scavenger (Methanol, MeOH). The results in Fig. 7a show that the presence of methanol completely hinders the photocatalytic degradation of sucrose, while silver nitrate partially obstructs the photocatalytic reaction. Therefore, it suggests that hydroxyl radical is the main active species for the photocatalytic degradation of sucrose over Ag/ZnO catalyst. The mechanism for the photocatalytic degradation of sucrose is depicted in Fig. 7b. First, the photoinduced electrons and holes are generated under UV irradiation. Then, the separation of the photoinduced electrons and holes occurs via the transportation of electrons on the surface of ZnO to metallic Ag nanoparticles allowing holes to generate hydroxyl radicals with H_2_O or OH^−^. In another way, electrons on Ag nanoparticles can also react with oxygen and form superoxide radicals. Therefore, both OH^.^ and O_2_^.^ cooperatively degrade the sucrose^28,29^.

### Role of Ag in the enhancement of photocatalytic activity

In this study, ZnO's photocatalytic activity was enhanced by modifying the ZnO surface with metallic Ag nanoparticles. The deposition of Ag on the ZnO surface aims to influence the interfacial charge transfer process. When ZnO absorbs UV light, the promotion of electrons from the valence band to the conduction band occurs, generating photoexcited electrons and holes crucial for the photocatalytic process, as illustrated in Fig. 7c. However, in the case of pure ZnO, most of the photoexcited charges undergo recombination. Only a fraction of photoexcited electrons and holes can reach the surface and perform the photocatalytic reaction. This results in high photoluminescence intensity of ZnO, as seen in Fig. 3b. The presence of Ag promotes the transfer of photoexcited electrons from the conduction band of ZnO since the Fermi level of Ag (− 4.26 eV)^26^ is lower than the conduction band of ZnO (− 4.19 eV), preventing the recombination of photoexcited electrons and holes. The quenching of the photoluminescence intensity in Ag/ZnO samples in Fig. 3b verifies this electron transfer process. Addition to the reduction of recombination rate, active species i.e. superoxide ions (O_2_^⋅−^) is generated on Ag nanoparticles. This process occurs when the electron transferred to Fermi level of Ag induces the shift in energy position towards more negative potential which is close to the Fermi level of ZnO^30^. Consequently, electrons on Ag surface can react with dissolved oxygen forming superoxide ions (O_2_^⋅−^) which participate in photodegradation of sucrose. Consequently, the photocatalytic activities of Ag decorated ZnO catalysts are much higher than that of pure ZnO. This interfacial electron transfer depends on the distribution of Ag on ZnO surface and the microstructure of ZnO. The increase in Ag content did not affect the distribution of Ag on ZnO surface but increased the aggregation of Ag into larger size nanoparticles. Therefore, the photocatalytic activity of all Ag/ZnO samples were comparable.

### Recyclability and photocatalytic degradation of condensate from sugar mills

**Figure 8:**
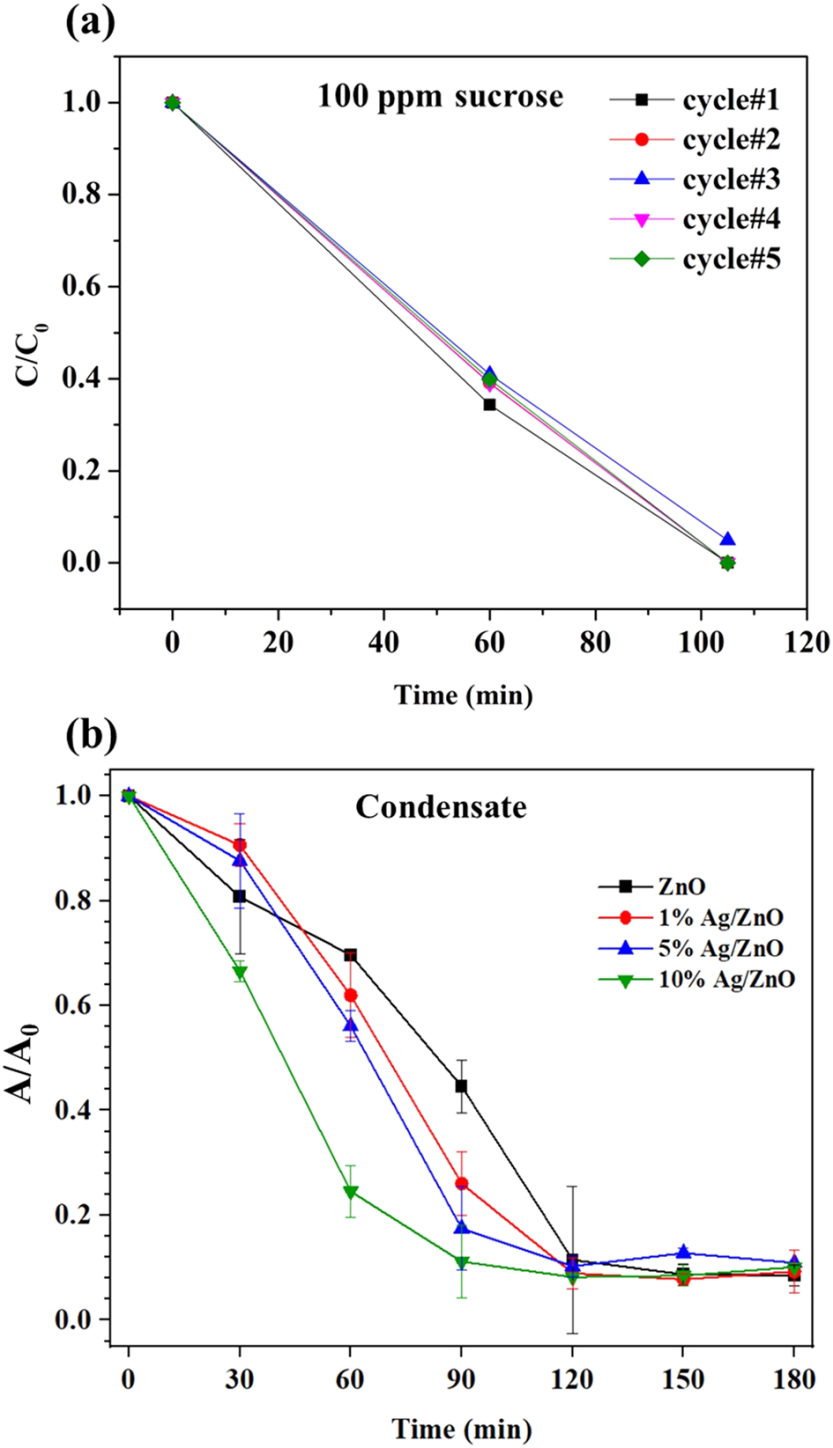
(**a**) Recyclability of 10%Ag/ZnO catalyst for 100 ppm sucrose degradation. (**b**) Degradation of sugar-contaminated condensate from the sugar mill.

Figure 8a presents that recyclability test of 10%Ag/ZnO. As can be seen, the declines of sugar concentration with time for all 5 cycles were almost superimposed, which indicates high stability of 10%Ag/ZnO. The Ag/ZnO catalysts were applied for the photodegradation of the condensate obtained from a juice evaporator process of the sugar mill. The initial concentration of condensate obtained from the colorimetric method was about 40 ppm. As revealed in Fig. 8b, ZnO and Ag/ZnO catalysts were able to degrade sugar in the condensate reaching approximately 90% degradation within 90 min. However, around 10% of residuals remained in the condensate due to the presence of large molecules like cellulose or carbohydrates that are difficult to degrade. It is seen that the degradation rates of Ag/ZnO catalysts were noticeably higher than that of ZnO. The estimated rate constants in degrading the condensate were 0.016, 0.020, 0.021, and 0.023 min^−1^ for ZnO, 1%Ag/ZnO, 5%Ag/ZnO, and 10%Ag/ZnO, respectively.

The condensate from the sugar mill was slightly turbid due to the presence of solid suspensions. The amount of solid suspension was determined by filtrating the condensate through 0.45 μm filter. The concentration of solid particles in the condensate was around 4 mg/100 mL. TOC analysis indicates that total carbon content in the condensate was about 38 mg/L which is higher than the carbon content in standard sucrose at 40 ppm (15 mg carbon/L). This suggests further that the condensate contained non-sugar components, supposedly cellulose. The concentration of sucrose, fructose and glucose in the condensate evaluated from HPLC were 17, 6 and 8 ppm, respectively (see the chromatograms in Fig. S1b in the Supplementary Information).

It is possible that the presence of solid suspensions in the condensate hindered the degradation rate by obstructing and absorbing UV light. The absorption of UV light by solid suspension in the condensate was investigated. Figure S2a in the Supplementary Information compares the spectrum of unfiltered condensate to that of 0.22 μm filtered condensate. Both UV absorption spectra of filtered and unfiltered condensate were similar with a peak at wavelength close to the maximum wavelength of UV lamps at 254 nm.

After the removal of solid suspension, the degradation rate of filtered condensate, shown in Fig. S2b in the Supplementary Information, revealed a significant improvement. However, the degradation rate of the filtered condensate was lower than that of sucrose solution due to the presence of remaining small size particles (less than 0.22 μm) in the filtered solution.

Dynamic light scattering (DLS) measurements showed that the particle size of solid suspension in the unfiltered and 0.22 μm filtered condensate was 996 and 82 nm, respectively (see Fig. S3 in the Supplementary Information).

The concentration of chloride in the condensate was about 15 ppm as measured by titration with 0.025 M AgNO_3_ standard solution using potassium chromate as an indicator (Mohr method). The presence of chloride ions in the condensate may cause the poisoning of the Ag containing catalyst. Therefore, the interference of chloride ion in the degradation of the condensate was studied by the addition of chloride ion into 40 ppm sucrose solution. As presented in the Supplementary Information Fig. S2b the chloride ions barely affected the photodegradation of sucrose by 10%Ag/ZnO.

## Conclusion

ZnO composite with Ag nanoparticles has been fabricated by two-step synthesis, i.e., sol–gel autocombustion followed by ion-impregnation. SEM images exhibited the change in the microstructure of ZnO with the addition of Ag nanoparticles according to the liquid sintering assisted by Ag nanoparticles. TEM images and EDS analysis confirmed the presence of Ag nanoparticles on the ZnO surface. The XRD patterns of Ag/ZnO samples indicated no incorporation of Ag into the ZnO lattice. In addition, the absorption edges of Ag/ZnO samples from DRS spectra exposed no difference from pure ZnO; therefore, both band gap energies were similar. A decrease in PL spectra intensities of Ag/ZnO compared with that of ZnO indicates the enhancement of photocatalytic activity by reducing the recombination of electron–hole pairs through the direct electron transfer process of photoexcited electrons from ZnO to metallic Ag. The results from Ag L3-edge XANES spectra demonstrated that the oxidation state of Ag nanoparticles in spent Ag/ZnO catalysts was zero suggesting the chemical stability of metallic Ag on the ZnO surface, which arises from the transfer of photoexcited electrons from ZnO. The HPLC and TOC analysis of degraded sucrose solution suggests that sucrose is degraded to glucose and fructose before degrading to carbon dioxide and water, confirming the mechanism of photocatalytic degradation. As evaluated from the scavenger tests, the crucial active species for sucrose degradation was hydroxyl radicals. The high performance of Ag/ZnO catalyst for high concentration (1000–2000 µg of carbon) of sucrose arises from the combination of well-calcined ZnO and robust metallic Ag nanoparticles. Ag/ZnO photocatalysts can effectively utilize for the degradation of sugar contaminated condensate. 10%Ag/ZnO can degrade the condensate for up to 90% within 90 min. The synthesis of Ag/ZnO in this study were facile and Ag/ZnO catalyst is promising for large-scale condensate treatment.

## Methods

### Synthesis of pure ZnO and Ag/ZnO composites

ZnO was prepared by the autocombustion sol–gel method. All reagents including zinc nitrate hexahydrate (Zn (NO_3_)_2_ × 6H_2_O), citric acid and poly (vinyl alcohol) (PVA, Mw = 130,000, 99% hydrolyzed) were used as received. Citric acid served as the chelating agent and fuel. The molar ratio of nitrates to citric acid was fixed at 3.4. PVA was used as a particle size-controlling agent. The amount of PVA was 20 wt% of the total weight of citric acid and PVA. First, PVA was dissolved in deionized (DI) water at ~ 100 °C. Once PVA was fully dissolved, the other reactants were added to the solution. The solution was continuously heated at 100–120 °C to evaporate water until the solution turned yellow and became viscous. By increasing the temperature, the thick-yellow solution started to decompose to obtain the brown solid. This brown precursor was ground and calcined in air at 550 °C for 2 h. After calcining, the white powder of ZnO was obtained.

Ag/ZnO composites were prepared by the ion impregnation method. The desired amount of AgNO_3_ was dissolved with a small amount of ethanol–water solution in a crucible before adding calcined ZnO powder. The mixture was sonicated for 15 min before calcining at 550 °C for 1 h in air. The amount of Ag varied from 1, 5, 10% by weight of ZnO denoted respectively as 1%AgZnO, 5%Ag/ZnO and 10%Ag/ZnO.

### Characterization of Ag/ZnO composites

The phase purity of calcined samples was characterized by XRD (Empyrean, Panalytical). Light absorption properties of the samples were studied by UV–vis Diffuse Reflectance spectroscopy (UV–vis DRS) performed on a Shimadzu spectrometer model UV-3101PC equipped with an integrating sphere and using BaSO_4_ as reference. The microstructure of composite particles was analyzed by scanning electron microscopy (miniSEM) (SEC, SNE-4500M). The presence of Ag nanoparticles on ZnO surface was investigated by transmission electron microscopy equipped with energy-dispersive spectroscopy (EDS) (TEM, FEI/Tecnai G^2^ 20s twin, Netherland). The oxidation state of Ag in fresh and spent Ag/ZnO samples was analyzed by X-ray Absorption Spectroscopy (XAS) conducted at BL8: XAS beamline at the Synchrotron Light Research Institute (SLRI), Thailand. The used Ag/ZnO sample was collected after completing the photocatalytic activity test. The used catalyst was separated from the aqueous solution by centrifuging and rinsed 2–3 times with DI water before drying in an oven at 60 °C overnight.

The room-temperature photoluminescence (PL) spectra of the ZnO and Ag/ZnO were obtained using a Shimadzu RF-5301PC Spectrofluorophotometer with a Xe lamp as the excitation source. The excitation wavelength used in the PL measurement was 350 nm.

The specific surface area of ZnO and Ag/ZnO samples were analyzed by nitrogen adsorption–desorption method using Microstrac BELSORP MAX. Prior to analyzing, the samples were degassing at 150 °C for 3 h under a vacuum pressure of 0.5 mmHg. The specific surface area was estimated by the BET method.

### Photocatalytic activity of Ag/ZnO composites

Photocatalytic activity of the catalyst was evaluated from the degradation of sucrose under 72 watts UV-C irradiation (2 lamps of 36 watts TUV PL-L Philips lamp, λ_max_ = 254 nm). The distance from lamps to solution was fixed at 10 cm. First, 40 mg of catalyst was dispersed in 30 ml (catalyst loading of 1.35 g/dm^3^) of diluted sucrose solution (100 or 200 ppm sucrose in deionized water) for 15 min using an ultrasonic bath (E 100H Elmasonic, Elma). The suspension was kept in the dark for 30 min to reach the adsorption–desorption equilibrium. Finally, the beaker containing the suspension was placed under the UV lamps with a distance of 10 cm from the top. During the irradiation, the solution was stirred continuously using a magnetic stirrer. 3 mL of the solution was withdrawn from the reactor every 15 or 30 min and was filtered through a 0.22 μm nylon filter membrane to remove solid catalyst. The total concentration of sucrose and its derivatives was determined by colorimetric method^31^. 2 mL of filtered solution was pipetted into a glass vial, followed by adding 1 mL of 5%w/w phenol aqueous solution and 5 mL of conc. H_2_SO_4_, respectively. In the presence of sucrose, a bright orange solution was obtained. The absorbance of the orange-solution was determined at 490 nm using UV–vis spectrophotometer (SPECORD 200 PLUS, Analytik Jena). The concentration of sucrose was evaluated from a calibration curve. The photocatalytic degradation rate was calculated by the following expression:

$$\mathrm{Degradation\; rate }(\mathrm{\%}) =\frac{{C}_{0}-C}{{C}_{0}} \times 100$$

where *C* and *C*_0_ are the sucrose concentration at specific time and at initial, respectively.

The degradation of sucrose was also confirmed by high performance liquid chromatography (HPLC) with refractive index detector (RID) and Agilent Hi-Plex Ca (DuO) column. Double-deionized water (ELGA, Ultra GE MK2) was used as a mobile phase. HPLC grade sugar standards (Sucrose were purchased from Ajax Finechem. Fructose and glucose were purchased from sigma-aldrich. The concentration of sugar was calculated from the calibration curve of standard solution.

Total organic carbon (TOC) analyzer (multi N/C 2100 sla, Analytik Jena) was employed in order to evaluate the amount of total organic carbon in the solution after the photocatalytic degradation for 0, 60, 105 and 150 min. The solution for TOC measurements was collected from the photocatalytic activity testing of 10% Ag/ZnO catalyst during photodegradation of 100 ppm sucrose solution.

### Scavenger experiments

The active species involved in the photocatalytic degradation of sucrose by 10%Ag/ZnO were investigated. AgNO_3_ (50 mmol) and methanol (5%v/v) were used as scavengers to capture electrons (e^−^), and hydroxyl radicals (OH^⋅^), respectively. The experiment was performed using 100 ppm sucrose solution and 1.35 g/dm^3^ catalyst. The solution was taken from the reaction at a specific time and was analyzed by HPLC.

### Reusability

The stability of the photocatalyst was evaluated by repeatedly performing the photocatalyst. 100 ppm of sucrose solution was initially photo-degraded by 1.35 g/dm^3^ of 10%Ag/ZnO. The sucrose solution drawn from the reaction was analyzed by colorimetric method at 0, 60 and 105 min. After the 1st cycle, 20 cm^3^ of 250 ppm sucrose solution was added into the mixture of catalyst and the residual solution creating the 100 ppm sucrose solution for the next cycle. The amount of catalyst was maintained the same throughout the reusability testing.

### Photocatalytic degradation of condensate from sugar mill

Ag/ZnO and ZnO catalysts were used in photodegradation of sugar-contaminated condensate obtained from a juice evaporator of the sugar mill (Mitr Phu Wiang sugar factory, Khon Kaen, Thailand). The same testing procedure with sucrose solution was employed for the condensate. The initial concentration of the condensate of about 40 ppm was determined by colorimetric method. The degradation rate was considered from the plot between A/A_0_ (A and A_0_ are Absorbance at specific time and at initial, respectively) instead of C/C_0_ since the condensate comprises sugars and non-sugar components like cellulose. The condensate was further analyzed by HPLC, TOC, and chloride titration. The chloride concentration was evaluated by titration with 0.025 M AgNO_3_ standard solution using potassium chromate as an indicator (Mohr method). The condensate was filtered through 0.45 and 0.22 μm nylon membranes. The solid residue on the membranes was collected and dried in an oven at 60 °C for 2 h to determine the amount of solid suspension. The particle sizes of solid suspension were measured by dynamic light scattering (Malvern Zetasizer nano ZS).

## Supplementary Information

Supplementary Figures.

## Data Availability

The datasets generated during and/or analyzed during the current study are available from the corresponding author on reasonable request.
