# Supplementary material for: Facile synthesis of robust Ag/ZnO composites by sol–gel autocombustion and ion-impregnation for the photocatalytic degradation of sucrose
Source: Sci Rep. 2023 Jul 27;13:12173. doi: 10.1038/s41598-023-39479-7 (PMC10374612; doi:10.1038/s41598-023-39479-7)
Supplement: Supplementary file 1 — Supplementary Figures. [file 41598_2023_39479_MOESM1_ESM.pdf]

Supplementary Information for

Facile synthesis of robust Ag/ZnO composites by sol-gel autocombustion and ion-impregnation for the photocatalytic degradation of sucrose

Lalita Buengkitcharoen[a,b], Sittipong Amnuaypanich[a,b], Suriyabhorn Naknonhan[b], Sirinuch Loiha[a,b], Nopbhasinthu Patdhanagul[c], Ammarika Makdee[d], Sujitra Amnuaypanich[a,b,*]

[a] Department of Chemistry and the Center of Excellence for Innovation in Chemistry (PERCH-CIC), Faculty of Science, Khon Kaen University, Khon Kaen 40002, Thailand

[b] Materials Chemistry Research Center (MCRC-KKU), Faculty of Science, Khon Kaen University, Khon Kaen 40002 Thailand

[c] Department of General Science, Faculty of Science and Engineering, Kasetsart University, Sakon Nakhon 47000, Thailand

[d] Synchrotron Light Research Institute, 111 University Avenue, Muang District, Nakhon Ratchasima 30000, Thailand

[*]Corresponding author: sujitra_kn@kku.ac.th (S. Amnuaypanich)

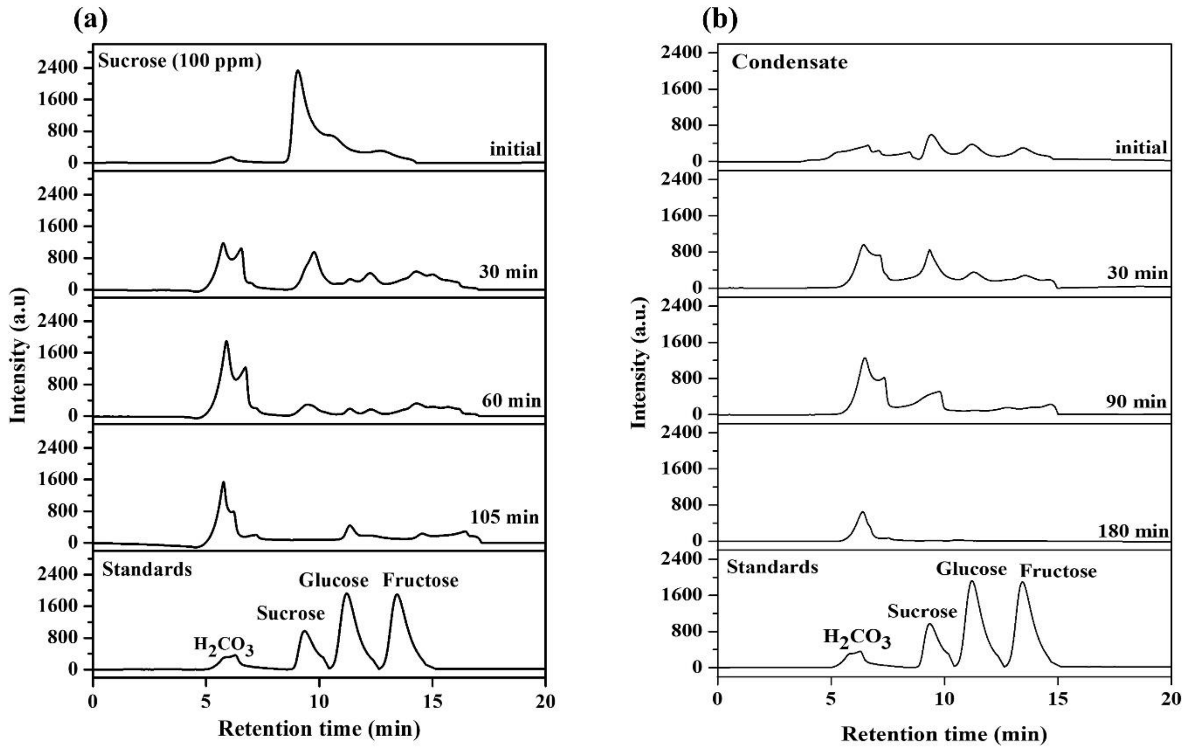

Figure S1. HPLC chromatograms of (a) photodegradation of 100 ppm sucrose by 10%Ag/ZnO and (b) photodegradation of condensate by 10%Ag/ZnO.

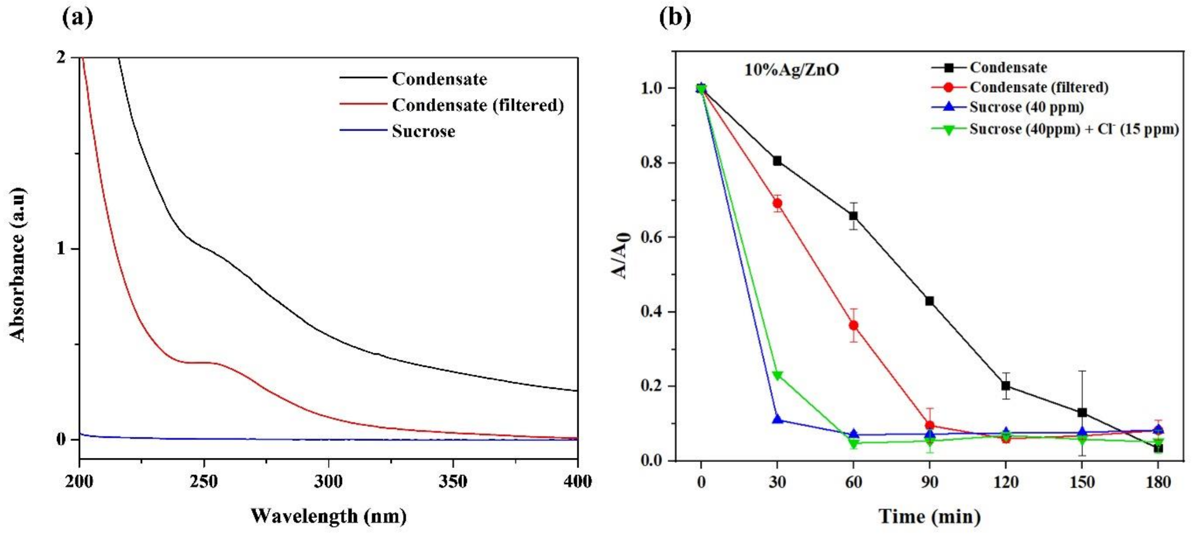

Figure S2. (a) UV absorption spectra of condensate, 0.22 μm filtered condensate, and sucrose (b) The photodegradation by 10%Ag/ZnO of condensate, 0.22 μm filtered condensate, sucrose, and sucrose with Cl^- ions.

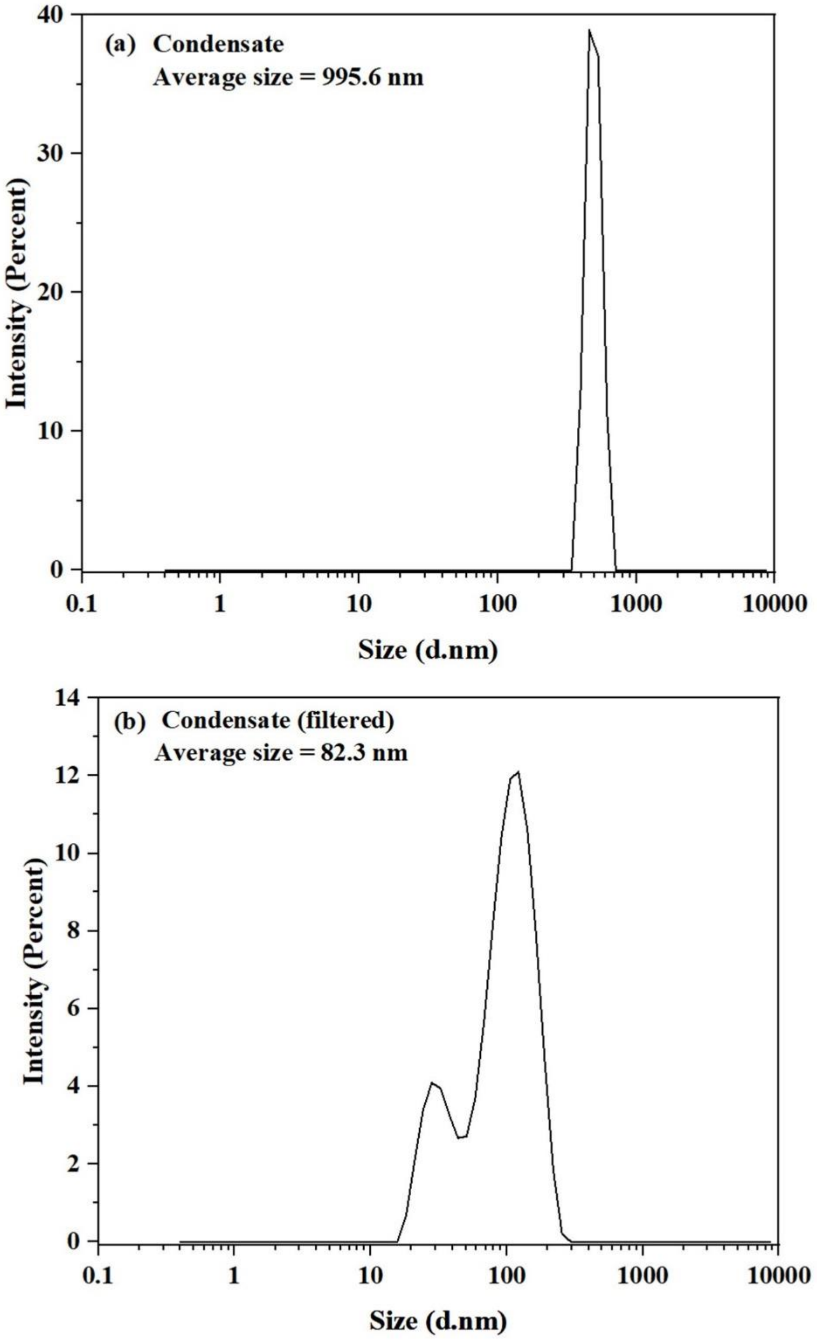

Figure S3. Particle size distribution from DLS of (a) condensate and (b) 0.22 μm filtered condensate.
